# ARABINOSE MITIGATE METABOLIC STRESS IN INFLAMMAGING

**DOI:** 10.1093/geroni/igae098.3635

**Published:** 2024-12-31

**Authors:** Brenda Landvoigt Schmitt, Patricia Silva da Pantoja, Brandt D Pence

**Affiliations:** University of Memphis, Memphis, Tennessee, United States; University of Memphis, Memphis, Tennessee, United States; University of Memphis, Memphis, Tennessee, United States

## Abstract

Arabinose (Ab) is a monosaccharide found in vegetables, fruits, and grains. It has been showed that Ab can manage blood sugar levels, improve gut health, potential anti-aging, antidepressant, antioxidants. Inflammaging is a chronic low graded inflammation commonly seen in aging, contribute significantly to age related disease. We aimed to investigate the impact of Ab on the gene expression of pro-inflammatory cytokines (IL-1β, IL-6, and TNF-α) which are implicated in inflammaging. Additionally, we investigate if the Ab impacted the bioenergetic cell metabolism using Seahorse methodology to assess mitochondrial oxidative phosphorylation (OxPhos) activity, measured as the oxygen consumption rate (OCR) and extracellular acidification rate (ECAR) mitigating inflammation induced by lipopolysaccharide (LPS). U937 cell line was cultured in RPMI media with Pen-Strep 1% and FBS 10%. RTq-PCR and mito-stress-test using Seahorse XF technology were performed. The data analysis was performed in Prism 9. Cells were pre-treated with Ab 30 min following the addition of LPS 3h incubation time at 37°C, 5% CO2 A dose response test was performed were the pre-treatment showed significative response with Ab 50uM in pro-inflammatory cytokines genes downregulating IL-1β, IL-6, and TNF-α. The OCR metabolic phenotype and maximal respiration, under stress conditions, was improved by pre-treatment with Ab in presence of LPS. Ab protected cell to increase ECAR even in presence of LPS. Overall, these results suggest an improvement on mitochondrial OxPhos capacity maintaining the glycolysis performance of monocytic cells under inflammatory stress offering potential benefits for managing inflammaging in older populations.

